# Psychometric properties of the Children’s Perception of Interparental Conflict Scale (CPIC), in a sample of Chilean adolescents

**DOI:** 10.3389/fpsyg.2026.1895075

**Published:** 2026-09-22

**Authors:** Cecilia Mayorga-Muñoz, José Luis Gálvez-Nieto, Karina Polanco-Levicán, Elisa Delvecchio, Ignacio Norambuena-Paredes, Felipe Quintano-Méndez

**Affiliations:** 1 Departamento de Trabajo Social, Universidad de La Frontera, Temuco, Chile; 2 Escuela de Psicología, Facultad de Ciencias Sociales y Educación, Universidad Santo Tomás, Temuco, Chile; 3 Department of Philosophy, Social Sciences and Education, University of Perugia, Perugia, Italy; 4 Programa de Doctorado en Ciencias Sociales, Universidad de La Frontera, Temuco, Chile; 5 Escuela de Psicología, Universidad Santo Tomás, Temuco, Chile

**Keywords:** Chilean adolescents, interparental conflict perception, perceived threat, psychometric properties, self-blame

## Abstract

**Introduction:**

Interparental conflict threatens adolescents’ emotional well-being and psychosocial adjustment. Within the cognitive-contextual framework, its developmental effects depend partly on adolescents’ appraisals of parental conflict, highlighting the need for culturally appropriate and psychometrically sound measures. This study evaluated the psychometric properties of the abbreviated Children’s Perception of Interparental Conflict Scale in Chilean adolescents.

**Methods:**

A cross-sectional, descriptive-correlational study included 435 students (*M* = 15.88 years, SD = 1.08) from eight public and subsidized schools in Chile, selected through stratified random sampling. Confirmatory factor analyses using the WLSMV estimator compared first-order, second-order, and two-factor models while accounting for the clustering of students within classrooms. Reliability was assessed using McDonald’s omega (ω) and Cronbach’s alpha (α).

**Results:**

The retained model (M3) comprised 35 items distributed across nine correlated first-order factors, following the removal of Item 34 because it produced a Heywood case. M3 showed excellent fit: WLSMV χ^2^ (524) = 701.23, *p* < 0.001, CFI = 0.974, TLI = 0.971, RMSEA = 0.029, and SRMR = 0.074. All standardized loadings were significant (*p* < 0.001), ranging from 0.524 to 0.946. Scalar measurement invariance across sex was supported (CFI = 0.002, RMSEA = −0.002, SRMR = 0.000). Across the nine first-order factors, reliability ranged from ω = 0.811 to 0.925 and α = 0.685–0.839. The second-order model produced an inadmissible solution, whereas the alternative two-factor models showed substantially poorer fit.

**Discussion:**

The findings support the internal structure and reliability of the 35-item, nine-factor CPIC for Chilean adolescents and its use in educational, clinical, and research settings.

## 1 Introduction

Couple dissolution constitutes a phenomenon of psychosocial and ecological dimensions with implications for the family as a whole (Escapa, 2017; Romero Antola, 2020). It demands the reorganization of boundaries and roles, rendering the coparental relationship a central element in children’s well-being (Mayorga-Muñoz et al., 2023; Iraurgi et al., 2008), given the influence that relationships within the family exert on children’s emotional well-being (Delgado et al., 2016). Accordingly, the study of interparental conflict is particularly relevant when the aim is to understand how children perceive, interpret, and respond to parental disputes.

Interparental conflict refers to disagreements, arguments, and disputes occurring between parents within a marital relationship (Fincham and Grych, 2001). This definition highlights that interparental conflict entails negative interactions between parents, such as verbal or physical aggression, hostility, and nonverbal expressions of anger (Fincham and Grych, 2001; Timmons and Margolin, 2015), and prolonged disparagement between both parents (López Larrosa et al., 2012), generating detrimental effects on the family. This conflict can be understood both in terms of its observable properties and the cognitive appraisal that children make of it, dimensions that are detailed further below together with the instrument used to measure them.

Although all marriages exhibit some degree of conflict, it is unlikely that all its manifestations are stressful for children (Grych and Fincham, 1990). Rather, children’s exposure to interparental conflicts that are frequent, intense, and poorly resolved increases the risk of adjustment problems (Huang et al., 2024; Olatunji and Idemudia, 2021), affecting both their mental and emotional health and their behavior, directly influencing the sense of security they experience within the family system and undermining the parent–child bond (van Dijk et al., 2020).

Evidence indicates that high-conflict family dynamics can significantly affect adolescents’ mental health, increasing their levels of anxiety and depression (Asanjarani et al., 2022). According to Hess (2022), continued exposure to interparental conflict is associated with a higher incidence of emotional and behavioral problems in children; as well as with deterioration in behavioral functioning, particularly when perceptions of threat and self-blame are elevated (Fosco and Feinberg, 2015; Giani et al., 2025), underscoring the need for early intervention. Similarly, research by O’Hara et al. (2022) shows that the impact of divorce on children’s mental health varies according to the coping strategies adopted and the degree of persistent parental conflict. This impact is further conditioned by the moderating role of the dimensions of conflict, gender, age, and the coping strategies employed by children, as well as by the emotional insecurity that observing conflict between one’s parents may entail (Chiang et al., 2024; Olatunji and Idemudia, 2021), which accounts for the short- and long-term detrimental effects that various studies have documented in the physiological, behavioral, emotional, and cognitive responses of children and adolescents (Zhao et al., 2022; Giraldo Vanegas, 2020; López-Larrosa et al., 2019). These findings support the need for instruments capable of capturing not only the presence of conflict but also its perceived meaning and impact from the child’s perspective.

Some systemic studies reveal that parents who experience frequent conflicts and fail to resolve them constructively tend to triangulate their children, forming coalitions with them against their partner (Koçak et al., 2024). This generates incompatibility and blurs the boundaries between the interparental subsystem and the parent–child subsystem, constituting a greater threat and fostering maladjustment in adolescents, which is associated with internalizing and externalizing behavior problems (Fosco and Bray, 2016; Koçak et al., 2024). Therefore, even after the couple’s relationship has ended, both parents must continue to be responsible for meeting their children’s needs and must cultivate an environment conducive to emotional, cognitive, and social learning (Garriga and Pennoni, 2022; Manrique de Lara Suárez et al., 2018). When couples relate to each other with kindness, trust, and understanding, they are able to manage and resolve their conflicts constructively, thereby providing children with feelings of security and well-being, whether they live together or apart (Fariña et al., 2020).

To explain how conflicts can affect child development, multiple conceptual models have been developed (Grych et al., 2003). The measurement of interparental conflict has typically involved assessing parents’ perceptions of their own behavior during such disputes (Reese-Weber and Hesson-McInnis, 2008). Other parent-report instruments have been reviewed by Fariña et al. (2020), including the Conflict and Problem-Solving Scales (Kerig, 1996), the Family Environment Scale (Moos, et al., 1984), and the Divorce-Separation Adjustment Questionnaire (Yárnoz Yaben and Comino González, 2010).

The measurement of conflict has also been directed toward children, through instruments such as the Children’s Perception of Interparental Conflict Scale (Grych et al., 1992) and the Interpersonal Conflict Questionnaire (Laursen, 1993). In addition, the Spanish adaptation of the CPIC (Iraurgi et al., 2008) is used, which is the version reported in the present study and is grounded in the cognitive-contextual model of Grych and Fincham (1990).

Specifically, the Cognitive-Contextual Model (Grych and Fincham, 1990) defines interparental conflict as a stressor that leads the child to attempt to understand and cope with it, proposing that the impact of parental conflict on children may be mediated by cognitive, emotional, and physiological factors (Crockenberg and Langrock, 2001; Cummings and Davies, 1994; Grych and Fincham, 1990; Katz, 2001). It further proposes a distinction between the primary and secondary processing that children undertake in response to interparental conflict (Iraurgi et al., 2008).

In this regard, the model suggests that, through primary processing, the child first becomes aware that a stressful event is taking place and experiences an initial affective reaction. During this stage, children appraise whether the conflict represents a direct threat to their safety or well-being and whether it has direct implications for them, which leads to secondary processing, a more elaborate stage during which the child attempts to understand why the conflict is occurring and how he or she should respond (Grych and Fincham, 1990).

To understand this process, it is necessary to consider, on the one hand, the characteristics of the conflict itself, including frequency, intensity, resolution, and stability, and, on the other, how children process this information that is, the degree of threat they experience, the attributions they make about it, their self-blame, and the perceived efficacy of their possible coping responses (Martínez-Pampliega et al., 2007). The CPIC is therefore theoretically grounded in the distinction between the properties of the conflict and the subjective appraisal made by the child, which renders it a particularly suitable instrument for assessing interparental conflict as perceived from a child and adolescent perspective.

### 1.1 Abbreviated Children’s Perception of Interparental Conflict Scale (CPIC, 36 items)

The Children’s Perception of Interparental Conflict Scale (CPIC) was developed by Grych et al. (1992) as a multidimensional self-report instrument for children and adolescents. The original version contained 49 items and recorded young people’s own evaluations of interparental conflict. Unlike instruments based on parental reports, the CPIC placed the child’s perspective at the center of the measurement process. Its development provided the foundation for assessing multiple aspects of interparental conflict within a single standardized instrument.

The original CPIC comprises nine first-order dimensions: frequency, intensity, resolution, stability, perceived threat, coping efficacy, triangulation, conflict content, and self-blame (Grych et al., 1992). These dimensions constitute the instrument’s primary measurement structure and allow scores to be examined at a differentiated level. Their simultaneous inclusion distinguishes the CPIC from instruments limited to the occurrence of parental disagreements. Consequently, the scale offers a multidimensional assessment of children’s and adolescents’ perceptions of interparental conflict (Moura et al., 2010).

Iraurgi et al. (2008) developed and validated the abbreviated 36-item Spanish-language version used as the basis for the present study. The abbreviated scale retained four items for each of the nine dimensions, preserving the multidimensional structure of the original instrument while reducing its administration time. This version was evaluated in 638 adolescents aged 11–17 years attending schools in Bizkaia, Spain. Their study provided the principal psychometric evidence supporting its use with Spanish-speaking adolescent populations.

In the CPIC-36, the nine dimensions are organized into three broader domains consistent with the structure of the instrument. Conflict properties comprises frequency, intensity, stability, and resolution; threat comprises perceived threat, coping efficacy, and triangulation; and self-blame comprises conflict content and self-blame (Iraurgi et al., 2008). This hierarchical organization permits the simultaneous examination of nine specific dimensions and three second-order factors. It also preserves the differentiated contribution of each subscale within the overall measurement model.

Each dimension is represented by four items, resulting in a balanced distribution throughout the instrument. Scores can be calculated for the nine specific subscales and, when supported by the measurement model, for the three broader domains. This scoring structure enables both detailed and parsimonious interpretations of adolescents’ responses to the scale (Iraurgi et al., 2008).

The validation of the abbreviated version produced an overall Cronbach’s alpha coefficient of 0.91 and acceptable internal consistency across its dimensions, with coefficients ranging from 0.63 to 0.82 (Iraurgi et al., 2008). The CPIC-36 also showed a correlation of 0.99 with the total score of the original version. Confirmatory factor analysis provided evidence consistent with the proposed multidimensional structure. These findings indicated that item reduction preserved substantial psychometric information while producing a shorter and more efficient instrument.

The CPIC-36 has subsequently been employed in studies involving adolescent populations in Spain. López Larrosa et al. (2012) administered the instrument to 510 students aged 11–18 years, whereas Zuñeda et al. (2016) used it with adolescents with and without child-to-parent violence. More recent research with Peruvian adolescents has also supported the usefulness of abbreviated CPIC measures. This evidence has progressively extended the application of the instrument to different Spanish-speaking settings (Pomahuacre-Carhuayal et al., 2023).

Despite these applications, psychometric evidence for the CPIC-36 remains limited among Chilean adolescents, where linguistic equivalence does not necessarily guarantee an adequate internal structure or reliable scores. Accordingly, the present study evaluates the psychometric properties of the abbreviated scale in this population. Specifically, it examines a correlated nine-factor model and a hierarchical model comprising conflict properties, threat, and self-blame. Reliability is also estimated for the nine first-order dimensions and the three second-order factors.

## 2 Materials and methods

This quantitative study employed a non-experimental, cross-sectional psychometric design (Ato et al., 2013; Wang and Cheng, 2020). The study was non-experimental because no variables or study conditions were manipulated. It was cross-sectional because all data were collected through a single administration of the instruments, without longitudinal follow-up. Its primary purpose was to examine evidence of validity based on the internal structure and the reliability of the abbreviated Children’s Perception of Interparental Conflict Scale in Chilean adolescents, following recommendations for instrument-validation studies (Boateng et al., 2018).

### 2.1 Participants

The final sample consisted of 435 high school students recruited from eight public and government-subsidized private educational establishments located in Temuco, Valparaíso, and Iquique, Chile. The adequacy of the sample size was evaluated considering the 36 items included in the instrument and the complexity of the confirmatory factor models. The final sample exceeded the commonly recommended minimum of 10 participants per item for factor-analytic studies (Boateng et al., 2018).

A stratified, two-stage sampling procedure was employed. In the first stage, educational establishments were selected according to city and administrative dependency. In the second stage, students were randomly selected from the official enrolment lists provided by the participating schools, according to previously established quotas.

The eligibility criteria were: (a) being enrolled in the second or third year of secondary education at one of the participating schools; (b) having written informed consent from a parent or legal guardian; (c) providing informed assent; and (d) completing the study questionnaire. Living with both parents was not an eligibility criterion. Students who did not provide the required consent or assent or who did not complete the questionnaire were not included in the analytical sample.

Participants ranged in age from 14 to 19 years (*M* = 15.88, SD = 1.08). The sample included 207 males (47.6%) and 228 females (52.4%). Regarding living arrangements, 220 participants (50.6%) lived with both parents, either alone or together with siblings or other relatives, whereas 215 (49.4%) reported other household configurations (see Table 1).

**TABLE 1:** Main characteristics of the sample.

Variables	Categories	*n* (%)
Sex	Male	47.6%
	Female	52.4%
Who do you live with?	Both parents	15.4%
	Both parents, siblings, and other relatives	35.9%
	Only with my mother	12.6%
	Only with my mother, siblings, and other relatives	27.4%
	Only with my father	1.8%
	Only with my father, siblings, and other relatives	2.8%
	Other relatives (siblings, grandparents, or aunts/uncles)	3.4%
	I live in a group home/residential facility	0.2%
	With a foster family	0.2%
	I live alone	0.2%
Nationality	Chilean	82.2%
	Other nationality	13.8%

### 2.2 Instruments

To fulfill the study’s objectives, a sociodemographic questionnaire was administered, designed to collect relevant information about the participants. This instrument included questions related to variables such as age, sex, educational level, type of educational establishment, and other contextual characteristics pertinent to the analysis. The information obtained allowed for the characterization of the sample and contextualized the results based on the students’ sociodemographic profiles.

Additionally, to assess children’s perception of interparental conflict, the abbreviated Spanish version of the Children’s Perception of Interparental Conflict Scale (CPIC), originally developed by Grych et al. (1992) and adapted by Iraurgi et al. (2008), is used. This scale aims to measure adolescents’ subjective perception of the conflict between their parents, encompassing various dimensions that capture both the characteristics of the conflict and its emotional and behavioral impact (see Supplementary material).

The version used in this study comprises 36 items across nine specific factors: intensity, frequency, stability, resolution, perceived threat, coping efficacy, triangulation, conflict content, and self-blame. These dimensions are organized around a structure that structures the instrument’s theoretical model: conflict properties (intensity, frequency, stability, and resolution), threat (perceived threat, coping efficacy, and triangulation), and self-blame (content and self-blame). The brief version used in this study consists of 36 items, distributed across nine subscales with four items each, which allows for a clear anticipation of the structure of the confirmatory factor analysis (CFA) model used to assess these dimensions. This structure reflects the approach of the cognitive-contextual model, allowing for a comprehensive understanding of the impact of parental conflict on children (Iraurgi et al., 2008).

A brief definition of the nine dimensions is provided below. Within the conflict properties domain, intensity assesses the perceived severity of parental disagreements, including expressions of hostility and verbal or physical aggression. Frequency refers to how often conflict episodes occur, while stability concerns their perceived persistence over time and the expectation that they will recur. Resolution evaluates whether parents can constructively resolve their disagreements or whether conflicts remain unresolved and continue to affect family interactions (Cummings and Davies, 2010; Iraurgi et al., 2008; Moura et al., 2010).

Within the threat domain, perceived threat assesses adolescents’ expectations that interparental conflict may lead to negative consequences, such as escalating aggression, parental separation, or personal involvement in the dispute. Coping efficacy refers to adolescents’ beliefs about their ability to manage, respond to, or reduce the effects of conflict. Triangulation evaluates the extent to which adolescents feel drawn into parental disagreements, pressured to support one parent, or placed in an intermediary position between them (Grych et al., 1992; Iraurgi et al., 2008).

Finally, within the self-blame domain, conflict content assesses whether adolescents perceive that parental disagreements concern their behavior, characteristics, or circumstances rather than issues exclusively related to the parental relationship. Self-blame evaluates the extent to which adolescents attribute the origin or continuation of interparental conflict to themselves. These dimensions capture adolescents’ causal interpretations of parental disagreements and their perceived personal responsibility for those experiences, including the guilt that may arise from such attributions (Iraurgi et al., 2008; Moura et al., 2010).

The abbreviated version of the Children’s Perception of Interparental Conflict Scale (CPIC-36; Iraurgi et al., 2008) demonstrated high reliability in the validation study conducted with a sample of 638 adolescents. The Cronbach’s alpha coefficient (α) for the total scale was α = 0.91, which indicates excellent internal consistency. The individual subscales also showed acceptable reliability coefficients, with values ranging from α = 0.63 to α = 0.82. Frequency (α = 0.82), resolution (α = 0.81), and self-blame (α = 0.80) subscales were particularly noteworthy. These results are comparable and, in some cases, even superior to those obtained in the original 49-item version. Likewise, the abbreviated version demonstrated a high degree of concordance with the full version (*r* = 0.99 for the total score), which supports its robustness as a reliable instrument for assessing children’s perceptions of interparental conflict from their perspective.

The confirmatory factor analysis of the CPIC-36 (Iraurgi et al., 2008) showed a good fit of the theoretical model, with GFI, AGFI, and NFI values of 0.84, 0.83, and 0.83, respectively. Although these indices are slightly below the conventional threshold of 0.90, they are considered acceptable in complex models. Furthermore, an RMSEA of 0.037 stands out, which indicates an excellent fit. These results support the construct validity of the instrument.

### 2.3 Procedure

The research was conducted in different stages, beginning with the random selection of two educational establishments for each type of administrative dependency in each of the cities included in the study. Subsequently, a formal invitation was sent to the principals and teachers of the selected establishments. In cases where an institution declined to participate, the reasons given were recorded, and a new establishment was randomly selected as a replacement. The exact numbers of schools and students initially invited, as well as those who declined or did not respond, were not systematically recorded. The final analytical sample consisted of 435 students from eight educational establishments.

It is important to note that the unit of analysis for this study was the students, whose selection was also carried out through a randomization procedure. To do this, each institution was asked to provide the official list of second- and third-year high school students. From these lists, participants were randomly selected according to previously established quotas until the required sample size was reached.

Once the corresponding institutional authorization was obtained, the delivery of informed consent forms to parents or guardians was coordinated with the school administration. Parents or guardians were required to provide written authorization for their children’s participation in the study. Then, before administering the instruments, the students’ informed assent was obtained in accordance with the dates and times previously agreed upon with each institution.

The research was approved by the Scientific Ethics Committee of the University of La Frontera, under Folio No. 027/20.

Data collection was conducted through an online, self-administered questionnaire hosted on the QuestionPro platform. The students were invited to participate through emails sent by the research team. These emails included information concerning the previously obtained parental consent, the corresponding informed assent, and the link to access the questionnaire. The information provided to participants outlined the objectives of the study, the voluntary nature of participation, the guarantees of confidentiality and data anonymity, the absence of associated risks, and their right to withdraw from the study at any time without negative consequences.

### 2.4 Data analysis

The first stage of the statistical analysis included the calculation of descriptive statistics, including measures of central tendency, dispersion, and asymmetry, as well as the evaluation of the univariate and multivariate normality of the items that make up the scale. To provide evidence of validity based on the internal structure, confirmatory factor analyses (CFAs) were conducted using Mplus software version 8.11 (Muthén and Muthén, 2017), based on a polychoric correlation matrix. For the estimation of the parameters, the Weighted Least Squares with Mean and Variance correction (WLSMV; Muthén and Muthén, 2017) method was used, which is recommended for the analysis of ordinal categorical variables. Because students were nested within classrooms, the clustered structure of the data was accounted for using TYPE = COMPLEX in Mplus, which provides corrected standard errors and model fit statistics. Regarding missing data, 23 of the 435 participants provided no valid responses and were excluded. Thus, the factor analyses included 412 participants, with the remaining missing data handled using WLSMV in Mplus.

To examine the latent structure of the Children’s Perception of Interparental Conflict Scale (CPIC-36), several competing CFA models were tested. First, a nine-factor correlated model was estimated, in accordance with the original structure proposed by Iraurgi et al. (2008). Alternative specifications were subsequently evaluated, including a second-order model and two-factor models representing the broader domains of conflict properties and threat. Models including the original 36 items and a 35-item specification excluding Item 34 were examined. The adequacy of the measurement model fit was evaluated using a battery of indices: the chi-square, the Comparative Fit Index (CFI), the Tucker-Lewis Index (TLI), and the Root Mean Square Error of Approximation (RMSEA). According to specialized literature, models presenting values greater than 0.90 for the CFI and TLI indices (Schumacker and Lomax, 1996), as well as values less than 0.08 for the RMSEA (Browne and Cudeck, 1993), are considered indicative of a good fit.

Measurement invariance across sex was subsequently examined for the retained measurement model using multigroup CFA with the WLSMV estimator. Given the ordinal nature of the items, configural and scalar invariance models were evaluated. The configural model assessed whether the same nine-factor structure was supported across groups, whereas the scalar model imposed equality constraints on factor loadings and thresholds across groups. Because a separate metric invariance model is not identified under this parameterization for categorical indicators estimated with WLSMV, the analysis proceeded directly from configural to scalar invariance. Measurement invariance was evaluated by examining changes in model fit indices between nested models, with particular attention to changes in CFI and RMSEA, together with the WLSMV-adjusted chi-square difference test (DIFFTEST). The clustered structure of the data was accounted for using TYPE = COMPLEX throughout the invariance analyses (Schumacker and Lomax, 2016).

The internal consistency of the retained CPIC measurement model was assessed for the nine first-order factors. Reliability was evaluated using McDonald’s omega (ω) and Cronbach’s alpha (α). McDonald’s omega was estimated from the standardized factor loadings and error variances derived from the retained first-order CFA model, consistent with a congeneric measurement model. In addition, 95% confidence intervals (CIs) were estimated for both reliability coefficients to account for sampling variability and provide information on the precision of the reliability estimates. These coefficients were interpreted according to the criteria proposed by Green and Yang (2015), as well as by Trizano-Hermosilla et al. (2021).

## 3 Results

### 3.1 Descriptive analysis

Table 2 presents the descriptive statistics for the 36 items of the scale, including means, standard deviations, skewness, and kurtosis. These statistics were examined to characterize item response patterns and identify potential floor or ceiling effects. They were not used as item-deletion criteria, as item retention was theoretically guided and evaluated through CFA. Among the items evaluated, Item 28 had the highest mean (*M* = 2.10, SD = 0.79) and exhibited low skewness (-0.18) and negative kurtosis (-1.39), indicating a relatively flat distribution. In contrast, Item 30 had the lowest mean (*M* = 1.14, SD = 0.39) and exhibited marked positive skewness (2.96) and high kurtosis (8.56), indicating a strong concentration of responses in the lower categories.

**TABLE 2:** Descriptive statistics.

Items	Mean	Std. dev.	Skewness	Kurtosis	K-S test
It1	1.60	0.77	0.84	−0.82	0.369
it2	1.46	0.71	1.23	0.05	0.411
it3	1.85	0.77	0.26	−1.28	0.251
It4	1.38	0.64	1.48	0.93	0.437
It5	1.45	0.70	1.24	0.12	0.413
It6	1.29	0.54	1.66	1.83	0.459
It7	1.53	0.73	1.01	−0.43	0.392
It8	1.38	0.68	1.50	0.80	0.443
It9	1.98	0.78	0.03	−1.34	0.21
It10	1.64	0.81	0.74	−1.08	0.363
It11	1.44	0.71	1.29	0.17	0.424
It12	1.30	0.59	1.84	2.24	0.467
It13	1.24	0.50	2.01	3.25	0.484
It14	1.40	0.65	1.38	0.63	0.426
It15	1.72	0.78	0.54	−1.17	0.316
It16	1.32	0.57	1.66	1.71	0.451
It17	1.63	0.75	0.73	−0.89	0.339
It18	1.21	0.52	2.39	4.73	0.498
It19	1.52	0.77	1.07	−0.47	0.401
It20	1.24	0.52	2.14	3.71	0.486
It21	2.07	0.78	−0.12	−1.36	0.216
It22	1.32	0.56	1.56	1.45	0.443
It23	1.34	0.62	1.62	1.38	0.447
It24	1.66	0.74	0.64	−0.91	0.318
It25	1.80	0.81	0.39	−1.36	0.295
It26	1.44	0.70	1.30	0.23	0.422
It27	1.42	0.69	1.37	0.44	0.425
It28	2.10	0.79	−0.18	−1.39	0.25
It29	1.33	0.60	1.66	1.61	0.453
It30	1.14	0.39	2.96	8.56	0.517
It31	1.30	0.60	1.82	2.11	0.465
It32	1.49	0.68	1.08	−0.12	0.389
It33	1.38	0.70	1.56	0.87	0.46
It34	1.44	0.68	1.26	0.26	0.417
It35	1.45	0.70	1.26	0.16	0.419
It36	1.47	0.70	1.15	−0.06	0.405

### 3.2 Confirmatory factor analysis

As shown in Table 3, M1 (36-item first-order model) provided acceptable overall fit; however, the solution was inadmissible because of a Heywood case involving Item 34. M2, which accounted for the clustered nature of the data using TYPE = COMPLEX, substantially improved the global fit indices but did not resolve the inadmissible solution associated with Item 34.

**TABLE 3:** Comparison of the competing confirmatory factor analytic models of the CPIC.

Model	Items	Structure	Cluster correction	χ ^2^(df)	CFI	TLI	RMSEA	SRMR	Model evaluation
M1	36	First-order (9 factors)	No	1416.98 (558)	0.936	0.928	0.061	0.082	Heywood case (Item 34)
M2	36	First-order (9 factors)	TYPE = COMPLEX	811.08 (558)	0.965	0.961	0.033	0.084	Heywood case (Item 34)
**M3**	**35**	**First-order (9 factors)**	**TYPE = COMPLEX**	**701.23 (524)**	**0.974**	**0.971**	**0.029**	**0.074**	**Best-fitting and admissible solution**
M4	35	Second-order (3 higher-order factors)	TYPE = COMPLEX	736.54 (550)*	0.970	0.967	0.030	0.079	Higher-order Heywood case
M5	36	Two correlated factors	TYPE = COMPLEX	–	0.875	0.867	–	–	Poor fit
M6	35	Two correlated factors	TYPE = COMPLEX	1448.09 (559)	0.870	0.862	0.062	0.144	Poor fit
M7	35	Two correlated factors	No	3012.04 (559)	0.806	0.793	0.103	0.142	Unacceptable fit

χ^2^, chi-square statistic; CFI, Comparative Fit Index; TLI, Tucker–Lewis Index; RMSEA, Root Mean Square Error of Approximation; SRMR, Standardized Root Mean Square Residual. TYPE = COMPLEX indicates that standard errors and model fit were corrected for the clustering of participants within classrooms.

*The M4 solution was inadmissible due to a higher-order Heywood case; therefore, the reported fit indices should be interpreted with caution. Bold values indicate the fit indices and model evaluation of the retained best-fitting and admissible model (M3).

To address this issue, Item 34 was removed and the first-order model was re-estimated. M3 (35-item first-order model with TYPE = COMPLEX) demonstrated the best overall performance, yielding excellent model fit (CFI = 0.974, TLI = 0.971, RMSEA = 0.029, SRMR = 0.074) and representing the only model that combined satisfactory global fit with an admissible measurement solution. Accordingly, M3 was retained as the final measurement model.

To evaluate whether a more parsimonious latent structure could adequately represent the data, M4 specified a second-order model based on the 35-item solution. Although this model exhibited acceptable global fit, it produced a higher-order Heywood case, indicating that the hierarchical structure was not psychometrically admissible.

Finally, M5, M6, and M7 examined alternative two-factor representations of the CPIC, in which the original dimensions were collapsed into the broader domains of Conflict Properties and Threat. All three models showed substantially poorer fit than M3, with lower CFI and TLI values and higher RMSEA and SRMR values. These findings indicate that reducing the multidimensional structure to two broad latent factors does not adequately reproduce the covariance structure of the data.

Table 4 presents the standardized factor loadings and squared multiple correlations for the retained first-order measurement model (M3). All standardized factor loadings were statistically significant and ranged from 0.524 (Item 33) to 0.946 (Item 1), indicating that all retained items showed meaningful associations with their respective latent factors. Most factor loadings exceeded the recommended threshold of 0.70, providing evidence of strong item-factor relationships. The squared multiple correlations (R^2^) ranged from 0.275 (Item 33) to 0.895 (Item 1), indicating that the retained items explained between 27.5% and 89.5% of the variance in their corresponding indicators. Overall, these findings provide additional support for the internal structure and reliability of the final 35-item first-order measurement model.

**TABLE 4:** Standardized factor loadings and explained variance for the final first-order measurement model (M3).

Factor	Item	Standardized factor loading (STDYX)	R^2^
Frequency	Item 7	0.876	0.767
	Item 11	0.888	0.789
	Item 14	0.838	0.702
	Item 27	0.839	0.704
Resolution	Item 1	0.946	0.895
	Item 15	0.887	0.787
	Item 21	0.669	0.448
Content	Item 2	0.608	0.369
	Item 16	0.878	0.771
	Item 22	0.894	0.799
	Item 29	0.902	0.814
Intensity	Item 3	0.905	0.819
	Item 9	0.702	0.493
	Item 24	0.886	0.785
	Item 28	0.564	0.318
Efficacy	Item 10	0.796	0.634
	Item 25	0.778	0.605
	Item 32	0.815	0.664
	Item 36	0.825	0.681
Threat	Item 4	0.928	0.861
	Item 12	0.904	0.817
	Item 18	0.931	0.867
	Item 33	0.524	0.275
Triangulation	Item 5	0.890	0.792
	Item 19	0.671	0.450
	Item 26	0.649	0.421
	Item 31	0.655	0.429
Self-blame	Item 6	0.874	0.764
	Item 13	0.903	0.815
	Item 20	0.890	0.792
	Item 30	0.805	0.648
Stability	Item 8	0.849	0.721
	Item 17	0.778	0.605
	Item 23	0.840	0.706
	Item 35	0.894	0.799

λ, standardized factor loading (STDYX); R^2^, squared multiple correlation. All standardized factor loadings were statistically significant (*p* < 0.001).

### 3.3 Factorial invariance

As shown in Table 5, measurement invariance of the CPIC-35 across sex was examined by comparing configural and scalar models. The configural model showed an acceptable fit to the data, χ^2^(1048) = 1586.049, *p* < 0.001, CFI = 0.955, TLI = 0.949, RMSEA = 0.051, 90% CI [0.045, 0.056], and SRMR = 0.088, supporting the same nine-factor structure among men and women.

**TABLE 5:** Measurement invariance of the CPIC-35 across sex.

Model	χ ^2^	df	*P*	CFI	TLI	RMSEA [90% CI]	SRMR	Δ CFI	Δ RMSEA	Δ SRMR	Δχ ^2^ (Δ df)	*P*
Configural	1586.049	1048	<0.001	0.955	0.949	0.051 [0.045, 0.056]	0.088	–	–	–	–	–
Scalar^1^	1621.106	1100	<0.001	0.957	0.953	0.049 [0.043, 0.053]	0.088	+0.002	**−0.002**	0.000	61.989 (52)	0.162

χ^2^, chi-square statistic; df, degrees of freedom; CFI, Comparative Fit Index; TLI, Tucker–Lewis Index; RMSEA, Root Mean Square Error of Approximation; CI, confidence interval; SRMR, Standardized Root Mean Square Residual; Δ, change relative to the preceding model. Models were estimated using the weighted least squares mean- and variance-adjusted estimator (WLSMV). Given the ordinal nature of the indicators, equality of factor loadings and item thresholds was tested jointly in the scalar model. The Δχ^2^ statistic corresponds to the robust chi-square difference test implemented in Mplus. Bold values indicate statistically significant results (*p* < 0.05).

^1^Given the ordinal nature of the indicators and the use of WLSMV estimation, factor loadings and item thresholds were constrained jointly in the scalar invariance model; therefore, a separate metric invariance model was not estimated.

Constraining factor loadings and item thresholds to equality across groups did not deteriorate model fit. On the contrary, the scalar model showed slightly improved fit indices, χ^2^(1100) = 1621.106, *p* < 0.001, CFI = 0.957, TLI = 0.953, RMSEA = 0.049, 90% CI [0.043, 0.053], and SRMR = 0.088. Changes in the approximate fit indices were negligible (ΔCFI = +0.002, ΔRMSEA = −0.002, and ΔSRMR = 0.000), and the robust chi-square difference test was nonsignificant, Δχ^2^(52) = 61.989, *p* = 0.162. Overall, these results support scalar measurement invariance of the CPIC-35 across sex.

Latent mean comparisons indicated only one statistically significant sex difference. Women showed a lower latent mean in Content than men (Mlatent = −0.264, SE = 0.127, *z* = −2.076, *p* = 0.038). Given that men constituted the reference group, this negative coefficient indicates that men reported higher levels of conflict content. No statistically significant latent mean differences were found for the remaining eight dimensions (*p* > 0.05).

### 3.4 Evidence of reliability

Next, the reliability of the retained 35-item CPIC was examined. As shown in Table 6, McDonald’s omega coefficients indicated adequate to excellent reliability across all nine first-order factors (ω = 0.811–0.925), whereas Cronbach’s alpha coefficients ranged from 0.685 to 0.839. Although alpha was slightly below 0.70 for Threat and Triangulation, their omega coefficients indicated adequate to excellent reliability. Overall, these findings support the internal consistency of the nine subscales in the retained first-order measurement model.

**TABLE 6:** Reliability estimates and 95% confidence intervals.

Factor	α	95% CI	ω	95% CI
Frequency	0.839	[0.803, 0.866]	0.919	[0.905, 0.934]
Resolution	0.797	[0.753, 0.834]	0.878	[0.839, 0.914]
Content	0.788	[0.755, 0.818]	0.896	[0.872, 0.920]
Intensity	0.738	[0.648, 0.792]	0.855	[0.831, 0.878]
Efficacy	0.774	[0.712, 0.816]	0.879	[0.852, 0.906]
Threat	0.685	[0.623, 0.730]	0.902	[0.867, 0.933]
Triangulation	0.699	[0.654, 0.750]	0.811	[0.770, 0.849]
Self-blame	0.789	[0.746, 0.820]	0.925	[0.901, 0.948]
Stability	0.815	[0.779, 0.843]	0.906	[0.891, 0.921]

α, Cronbach’s alpha; ω, McDonald’s omega; CI, confidence interval. The 95% CIs were estimated using cluster bootstrap for α and Monte Carlo simulation for ω. Omega was derived from the standardized CFA estimates of Model 3.

## 4 Discussion

The present study aimed to evaluate the psychometric properties of the CPIC among Chilean adolescents. The scale, comprising 35 items and nine correlated factors, demonstrated excellent fit and acceptable parameter estimates. This finding supports the multidimensional conceptualization proposed by Grych et al. (1992) and retained by Iraurgi et al. (2008). It is also consistent with recent evidence distinguishing different perceptions of interparental conflict among adolescents across diverse contexts (Kalantari et al., 2026; Pomahuacre-Carhuayal et al., 2023).

One noteworthy finding was the identification of a Heywood case associated with Item 34. Such improper solutions compromise model interpretation and may be related to estimation or specification problems (Kolenikov and Bollen, 2012). Removing this item yielded an admissible solution while retaining the resolution dimension with three indicators, thereby strengthening the evidence of structural validity without altering the scale’s conceptual foundation.

The retention of frequency, intensity, stability, and resolution supports the relevance of the observable properties of conflict within the cognitive-contextual model (Grych and Fincham, 1990). This distinction is substantively important because exposure to recurrent, intense, or poorly resolved conflict has been associated with aggression, depressive symptoms, and adolescent adjustment difficulties (Li et al., 2023; Asanjarani et al., 2022; Jiang et al., 2025). These associations may also vary according to individual reactivity and family emotional dynamics (Davies et al., 2022; Chiang et al., 2024).

The threat, coping efficacy, triangulation, conflict content, and self-blame factors represent the processes through which adolescents interpret the personal relevance, controllability, and causes of conflict (Grych et al., 1992; Iraurgi et al., 2008). Distinguishing among these appraisals is particularly relevant because they have been associated with externalizing problems, involvement in bullying, problematic smartphone use, and lower academic engagement (Qiao et al., 2025; Song et al., 2024; Lin and Zhu, 2025; Li and Han, 2025).

From a family perspective, the CPIC provides insight into how dynamics within the interparental subsystem influence children’s emotional security, communication, and well-being. Evidence indicates that parental and family characteristics shape the experience of conflict, particularly when adolescents become involved in parental disputes (Findley et al., 2026; Mills et al., 2021; Kanat et al., 2026). This assessment is also relevant in light of the intergenerational transmission of relational patterns and traumatic experiences (Ozturk, 2022, 2023), while complementing other family assessment instruments (Fariña et al., 2020).

The second-order model yielded acceptable overall fit indices; however, the alternative two-factor models showed substantially poorer fit. Accordingly, conflict properties, threat, and self-blame remain useful theoretical domains, but they do not constitute hierarchical scores supported by the present data. This finding confirms that the CPIC structure may vary according to developmental stage and cultural context (Reese-Weber and Hesson-McInnis, 2008; Iraurgi et al., 2008; Pomahuacre-Carhuayal et al., 2023).

Scalar measurement invariance across sex indicates that the CPIC-35 (see Table 5) assesses the nine dimensions equivalently in male and female adolescents, thereby supporting meaningful latent mean comparisons between groups. Only the Content dimension showed a statistically significant difference, with male adolescents reporting higher scores than females, suggesting that they were more likely to perceive parental disagreements as related to their own behavior or circumstances. No significant differences were observed in the other eight dimensions, indicating that perceptions of interparental conflict were generally similar across sex. Nevertheless, given that this was the only significant difference identified, it should be interpreted cautiously and replicated in future studies.

All factor loadings were statistically significant and ranged from 0.524 to 0.946, with most exceeding 0.70. Omega coefficients ranged from 0.811 to 0.925, with particularly strong values for self-blame, frequency, stability, and threat. These results equal or exceed the reliability estimates reported for several subscales in previous versions of the instrument (Iraurgi et al., 2008; Kalantari et al., 2026). The differences between omega and alpha coefficients, particularly for threat and triangulation, support the use of omega to estimate the internal consistency of congeneric measures (Green and Yang, 2015).

The availability of an appropriate measure for Chilean adolescents has implications for child-related policies, educational institutions, and psychosocial interventions (Martínez-Pampliega, 2008). Its application could facilitate the early identification of perceptions of conflict that may affect emotional well-being, overall mental health, and academic performance (Davinson-Pacheco et al., 2026; Norambuena-Paredes et al., 2025a). Furthermore, the resulting profiles could inform interventions targeting threat, self-blame, triangulation, and coping, while also supporting family counseling and referrals to specialized professionals (Chiang et al., 2024; Song et al., 2024; Li and Han, 2025; Fariña et al., 2020).

An important contribution of this study lies in foregrounding adolescents’ subjective perspectives, in contrast to instruments primarily focused on parental reports. This contribution aligns with a body of psychometric research conducted in Chilean educational and psychosocial contexts (Norambuena-Paredes et al., 2025b; Gálvez-Nieto et al., 2025; Polanco-Levicán et al., 2025, 2026; Klenner-Loebel et al., 2026; Troncoso-Tejada et al., 2026). By complementing adult reports with children’s own experiences, these findings expand the available evidence concerning the CPIC.

### 4.1 Limitations and implications for future research

This study has several limitations that should be considered when interpreting its findings. Although adolescents from different geographical areas and educational institutions participated, variability in CPIC scores according to specific sociodemographic characteristics was not examined. Variables such as socioeconomic status, parental educational attainment, cultural background, migration history, and family composition may influence how conflict is interpreted. Future studies should incorporate these characteristics and examine potential between-group differences.

Second, data were collected exclusively through a self-report instrument; therefore, responses may have been influenced by perceptual, recall, or social desirability biases. Although adolescents’ perspectives constitute the central focus of the CPIC, they should ideally be complemented with information provided by parents, caregivers, and professionals. Integrating quantitative and qualitative methods would enable a more in-depth understanding of the meanings that children attribute to interparental conflict.

Furthermore, potentially relevant family characteristics were not assessed, including custody arrangements, frequency of contact with the non-resident parent, communication between separated parents, and the time elapsed since parental separation. The child’s age at the time of the parental breakup was also not considered. Incorporating these variables would make it possible to examine how family configurations and co-parenting arrangements are associated with perceptions of threat, triangulation, and self-blame.

The cross-sectional design precluded the examination of the temporal stability of scores and changes in perceptions of conflict throughout adolescence. Moreover, it did not allow the predictive ability of the dimensions in relation to emotional well-being, mental health, or academic performance to be established. Longitudinal studies are needed to assess test–retest reliability, longitudinal invariance, and temporal relationships between the CPIC dimensions and various indicators of psychosocial adjustment.

Finally, the removal of Item 34 and the inadmissible solution obtained for the second-order model warrant caution when interpreting hierarchical scores. Future studies should replicate the correlated 35-item structure in independent samples and examine the stability of its parameter estimates. It would also be pertinent to evaluate alternative factor models and provide evidence of convergent, discriminant, and predictive validity without presupposing the existence of stable higher-order factors.

## 5 Conclusion

The Interparental Conflict Scale from the Children’s Perspective (CPIC-36) demonstrated adequate psychometric properties in Chilean adolescents, confirming its structural validity and internal consistency across the nine dimensions it assesses. This finding provides relevant evidence for the Latin American context and strengthens the cross-cultural utility of the instrument. The study offers a valid and reliable tool to assess children’s perceptions of interparental conflict in clinical, educational, and research settings, contributing both to the early detection of risk factors and to the design of psychosocial interventions aimed at mitigating the negative impact of conflict on the mental health of children and adolescents.

## Data Availability

The raw data supporting the conclusions of this article will be made available by the authors, without undue reservation.
