# Supplementary material for: Psychometric properties of the Children’s Perception of Interparental Conflict Scale (CPIC), in a sample of Chilean adolescents
Source: Front Psychol. 2026 Sep 22;17:1895075. doi: 10.3389/fpsyg.2026.1895075 (PMC13638647; doi:10.3389/fpsyg.2026.1895075)
Supplement: Supplementary file 1 [file Supplementary_file_1.DOCX]

**Material suplementario / Supplementary Material**

Se proporciona el siguiente material suplementario para acompañar nuestro manuscrito/ The following supplementary material is provided to accompany our manuscript:

1. Escala de Conflicto Interparental desde la Perspectiva de los Hijos (CPIC)/ Children’s Perception of Interparental Conflict Scale (CPIC):

Instrucciones/ **Instructions:**

En todas las familias hay ocasiones en las que los padres no se llevan bien. Cuando los padres discuten los/as niños/as pueden experimentar diversos sentimientos. Queremos saber lo que sientes cuando tus padres discuten. Si tus padres no viven en la misma casa, piensa en las ocasiones en las que están juntos y discuten o bien recuerda el tiempo que ambos vivían contigo.

A continuación, tienes unas frases en las que debes indicar si son Verdad, Casi verdad o Falso, marcando el círculo que corresponda. / **In all families, there are times when parents do not get along. When parents argue, children may experience different feelings. We want to know how you feel when your parents argue. If your parents do not live in the same household, think about the occasions when they are together and argue, or remember the time when both of them lived with you.**

**Below are some statements. Please indicate whether each one is True, Almost true, or False by marking the corresponding circle.**

Versión original en español / **Original version in Spanish**

|  |  | Verdad | Casi verdad | Falso |
| --- | --- | --- | --- | --- |
| 1 | Mis padres hacen las paces después de discutir. | ☐ | ☐ | ☐ |
| 2 | Mis padres suelen discutir sobre cosas que hago en el colegio. | ☐ | ☐ | ☐ |
| 3 | Mis padres se enfadan(enojan) mucho cuando discuten. | ☐ | ☐ | ☐ |
| 4 | Cuando mis padres discuten siento miedo. | ☐ | ☐ | ☐ |
| 5 | Me siento atrapado entre mis padres cuando discuten. | ☐ | ☐ | ☐ |
| 6 | Yo soy el culpable cuando mis padres discuten. | ☐ | ☐ | ☐ |
| 7 | Quizá ellos no sean conscientes de que me doy cuenta, pero mis padres discuten mucho. | ☐ | ☐ | ☐ |
| 8 | Mis padres discuten porque no son felices juntos. | ☐ | ☐ | ☐ |
| 9 | Mis padres discuten de forma tranquila y calmada cuando no están de acuerdo. | ☐ | ☐ | ☐ |
| 10 | No sé qué hacer cuando mis padres discuten. | ☐ | ☐ | ☐ |
| 11 | Mis padres se faltan al respeto incluso cuando yo estoy delante. | ☐ | ☐ | ☐ |
| 12 | Cuando mis padres discuten me preocupa lo que pueda ocurrirme. | ☐ | ☐ | ☐ |
| 13 | Cuando mis padres discuten suele ser culpa mía. | ☐ | ☐ | ☐ |
| 14 | A menudo veo a mis padres discutir. | ☐ | ☐ | ☐ |
| 15 | Mis padres suelen encontrar una solución después de discutir. | ☐ | ☐ | ☐ |
| 16 | Las discusiones de mis padres suelen ser sobre algo que he hecho yo. | ☐ | ☐ | ☐ |
| 17 | Las razones por las que discuten no cambian nunca. | ☐ | ☐ | ☐ |
| 18 | Cuando mis padres discuten temo que algo malo me ocurra. | ☐ | ☐ | ☐ |
| 19 | Mi madre quiere que le apoye cuando ella y mi padre discuten. | ☐ | ☐ | ☐ |
| 20 | Aunque ellos no lo admitan sé que mis padres discuten por mi culpa. | ☐ | ☐ | ☐ |
| 21 | Mis padres se hacen amigos enseguida después de discutir. | ☐ | ☐ | ☐ |
| 22 | Mis padres suelen discutir sobre cosas que he hecho yo. | ☐ | ☐ | ☐ |
| 23 | Mis padres discuten porque realmente no se quieren. | ☐ | ☐ | ☐ |
| 24 | Mis padres gritan cuando discuten. | ☐ | ☐ | ☐ |
| 25 | Cuando mis padres discuten no puedo hacer nada para evitarlo. | ☐ | ☐ | ☐ |
| 26 | Creo que debo apoyar a uno de los dos cuando mis padres riñen (pelean). | ☐ | ☐ | ☐ |
| 27 | Es habitual escuchar a mis padres quejarse y reñir (pelear) mientras andan por la casa. | ☐ | ☐ | ☐ |
| 28 | Mis padres casi nunca gritan cuando discuten. | ☐ | ☐ | ☐ |
| 29 | Mis padres a menudo discuten cuando hago algo mal. | ☐ | ☐ | ☐ |
| 30 | Mis padres me echan la culpa cuando discuten. | ☐ | ☐ | ☐ |
| 31 | Mi padre quiere que le apoye cuando él y mi madre discuten. | ☐ | ☐ | ☐ |
| 32 | Cuando mis padres discuten no puedo hacer nada para sentirme mejor. | ☐ | ☐ | ☐ |
| 33 | Cuando mis padres discuten temo que vayan a divorciarse. | ☐ | ☐ | ☐ |
| 34 | Mis padres siguen riñendo (peleando) incluso después de haber discutido. | ☐ | ☐ | ☐ |
| 35 | Mis padres discuten porque no saben cómo llevarse bien. | ☐ | ☐ | ☐ |
| 36 | Cuando mis padres discuten no escuchan nada de lo que digo. | ☐ | ☐ | ☐ |
